# Inner Leaf Gel of *Aloe striata* Induces Adhesion-Reducing Morphological Hyphal Aberrations

**DOI:** 10.3390/jof4010023

**Published:** 2018-02-08

**Authors:** Gloria Wada, Michael Vincent, Marcia Lee

**Affiliations:** 1 Department of Microbiology, Miami University, Oxford, OH 45056, USA; leemr@miamioh.edu; 2 Department of Biology, Miami University, Oxford, OH 45056, USA; vincenma@miamioh.edu

**Keywords:** *Aloe*, adhesion, antifungal, *Aloe striata*, germination, aberrations, *Fusarium*, *Paecilomyces*

## Abstract

Fungi, particularly molds that are cosmopolitan in soils, are frequent etiologic agents of opportunistic mycoses. Members of the *Fusarium solani* and *Fusarium oxysporum* species complexes are the most commonly implicated etiologic agents of opportunistic fusarial infections in mammals, while *Paecilomyces variotii* is one of the most frequently encountered *Paecilomyces* species in human infections. Prevention and treatment of these mycoses are problematic because available antimycotics are limited and often have toxic side effects. Popular folk medicines, such as the inner leaf gel from *Aloe* spp., offer potential sources for novel antimycotic compounds. To screen for antifungal properties of *Aloe striata*, we treated conidia of three strains each of *F. solani*, *F. oxysporum*, and *P. variotii* with homogenized and filtered inner leaf gel. Exposure to gel homogenates caused minimal inhibition of conidial germination in tested strains. However, it significantly increased the frequency of hyphal aberrations characterized by increased hyphal diameters that resulted in intervals of non-parallel cell walls. Non-parallel cell walls ostensibly reduce total hyphal surface area available for adhesion. We found a significant decrease in the ability of aberrated *P. variotii* hyphae to remain adhered to microscope slides after repeated washing with reverse osmosis water. Our results suggest that treatment with *A. striata* contributes to a decrease in the adhesion frequency of tested *P. variotii* strains.

## 1. Introduction

Due to a limited number of non-toxic and effective antimycotic drugs as well as the increasing number of emerging human fungal pathogens, it is imperative that novel antifungal treatments be identified. Filamentous fungi, such as members of the genera *Fusarium* and *Paecilomyces*, that were not considered human pathogens over 30 years ago, have been increasingly recognized as emerging pathogens [1,2,3,4,5]. *Paecilomyces* spp. were previously only regarded as contaminants often encountered in food and raw materials such as cereals, nuts, cheeses, fruits, and seeds that contain oils [1]. However, in recent years, they have been implicated in an array of opportunistic human infections [1,2,6,7,8]. *Paecilomyces variotii* is one of the most commonly encountered *Paecilomyces* species in human infections, including onychomycosis, pneumonia, sinusitis, otitis media, osteomyelitis, and hyalohyphomycosis [8,9,10,11,12,13,14]. Similarly, *Fusarium* species have become increasingly common etiologic agents in invasive disseminated fungal infections in immunocompromised individuals such as burn victims as well as human immunodeficiency virus (HIV) and neutropenic cancer patients [3,5,15,16,17,18,19,20,21]. They are also implicated in localized mycoses such as ocular infections, onychomycosis, and cutaneous mycoses [15,22,23,24,25,26,27]. Members of the *Fusarium solani* species complex are the most frequently implicated etiologic agents in invasive fusariosis in humans and a diversity of other animals, including dogs and turtles [28,29]. In addition to animals, members of the genus *Fusarium* also cause infections in plants. *Fusarium oxysporum* causes vascular wilt in economically important crops such as bananas and tomatoes. It single-handedly caused the widespread loss of the Gros Michel banana cultivar, the predominant banana cultivar used worldwide before 1960, in South America between 1940 and 1960 [30].

In the life cycles of filamentous fungi, such as *F. solani*, *F. oxysporum,* and *P. variotii*, germination is an important virulence factor because it marks the beginning of a new cycle of fungal development. The ability to germinate, form hyphae, and ultimately branch is important for the persistence of fungi in the vegetative stage of development, the stage when fungi can colonize hosts. It is also early in this stage that fungal hyphae can significantly contribute to virulence [31,32]. Branching increases the number of apices available to thigmotrope, to contact sense, and, subsequently, to re-direct themselves into openings associated with topographical changes in the environment. Since germination and other physiologically essential events of early fungal development are necessary for successful adherence to host substrate and subsequent invasion and colonization, it is important to find new antimycotic compounds that target the early stages of filamentous fungal development.

Plants are potential sources of compounds that inhibit germination and/or early post-germination developmental processes. Singh et al. [33] found that alkaloid compounds, dehydroxorydalmine and oxyberberine, extracted from the plant *Argemone mexicana* inhibited spore germination in five plant pathogenic fungi including *Fusarium udum*. In addition to germination, plant-derived compounds are implicated in the regulation of branching in fungi. In the absence of a plant host, spores from fungal genera *Gigaspora* and *Glomus* germinate, fungal growth is retarded, and branching frequency decreases [34]. Exudate from the plant *Lotus japonicus* promotes fungal branching in arbuscular mycorrhizal fungi [35]. Furthermore, Kashina et al. found that extracts from the plant *Flammulina velutipes* inhibit the adhesion of two dimorphic fungi, *Sporothrix schenkii* and *Candida albicans*, to epithelial cells [36].

Although *Aloe* inner leaf gel has been used in folk medicine since approximately 1500 BCE, most research has focused on *Aloe* species’ ability to reduce mycelial growth [37]. Exposure to inner leaf gel filtrates from *Aloe barbadensis* caused reduced fungal colony diameters in *Botrytis gladiolorum*, *F*. *oxysporum* f.sp. *gladioli*, *Heterosporium pruneti* and *Penicillium gladioli*, *Aspergillus* spp., *Cladosporium herbarum* and *Fusarium moniliforme*, *Trichophyton* spp., and *Epidermophyton floccosum* [38,39,40]. Most investigations into the antifungal effects of *Aloe* involve domesticated *A. barbadensis* and exclude other *Aloe* spp. Furthermore; few investigations have studied the specific effects of *Aloe* species on early fungal development and function. Therefore, *Aloe* spp. appear to be promising sources of novel compounds to disrupt germination and overall growth in filamentous fungi. To determine whether *A. striata* inner leaf gel possesses antifungal properties against early hyphal development, our study examines the effect of *Aloe striata* gel on the germination frequency of conidia from three strains each of *F. solani*, *F. oxysporum*, and *P. variotii*. We also observed hyphal aberrations in germinated *A. striata* treated fungi. Using adhesion assays, we tested the specific hypothesis that *A. striata* induces hyphal aberrations characterized by intervals of non-parallel cell walls along the length of hyphae that contribute to a decrease in the ability of fungi to adhere to substrate.

## 2. Materials and Methods

*Aloe striata* plants, originally from South Africa, were deposited in Krohn Conservatory (Cincinnati, OH, USA) where we obtained them. Plants were grown in a fungicide-free room in the Miami University greenhouse for at least three years before gel harvest. A voucher for these *A. striata* plants (Marcia Lee s.n., MU 258172, Figure S1) was deposited in the Willard Sherman Turrell Herbarium at Miami University (Oxford, OH, USA). Inner gel, aseptically removed from harvested leaves, was homogenized and sequentially filtered through 300 µm nylon mesh, 30 µm nylon mesh, 5 µm durapore, and 0.2 µm Media-Kap^®^ pore-size filters (Cole-Parmer, Vernon Hills, IL, USA) to obtain sterile, low-viscosity (ca. 1.00 centipoise) filtrates. The harvested leaves were 32.90 ± 7.54 × 7.07 ± 1.26 cm (length × width) (mean ± SD). The average yield of 0.2 µm filtrate per mL extracted inner leaf gel was 57.3% of total extracted gel. The *A. striata* filtrates were lyophilized and stored at 4 °C for future use. The lyophilized filtrates were rehydrated immediately before use.

We initially screened three strains each of *P. variotii*, *F. oxysporum,* and *F. solani*, which are referred to as Pv19, Pv06, and Pv23, respectively (Table 1). Fusarial strains are referred to as Fo24, Fo57, Fo69, Fs20, Fs53, and Fs02 (Table 1). All *Fusarium* strains were obtained from the Agricultural Research Service (ARS) Culture Collection located at the National Center for Agricultural Utilization Research in Peoria, IL, USA. We chose these strains because they are easy to culture and they consistently generate characteristically conidiating mycelia for their respective species. Furthermore, Pv19 and all of the *Fusarium* strains have portions of their genome already sequenced. This may become beneficial in the future to investigate *Aloe* inner leaf gel’s effect on fungi at the molecular level [3,41]. Stock cultures of the aforementioned fungi from −80 °C were inoculated onto four potato dextrose agar (PDA) slants. Cultures were grown at 25 °C until they were visibly mature with copious amounts of conidia. For each strain, 2 or 3 of the 4 slants that had characteristically conidiating mycelia were used for conidial harvest. Conidia were harvested from slants by flooding the slants with 10 mL of sterile 0.85% saline. The mycelial surface of each slant was gently disturbed with glass Pasteur pipettes. The resultant mixture of conidia and hyphal fragments were transferred to sterile vortex tubes. After the transfer, the suspensions were left untouched for 15–20 min to allow hyphal particles to settle to the bottom of the tube while the fungal conidia remained suspended in the upper layer of the solution. After 15–20 min, the upper homogenous top layer was transferred into Falcon conical tubes. The reclaimed conidial suspension was then centrifuged at 694× *g* for 15 min. The resultant supernatant was discarded and the pellet was washed by adding approximately 7 mL of reverse osmosis water (ROH_2_O), vortexed, and centrifuged at 694× *g* for another 15 min. The washing step was repeated once more. After the second washing, 2 mL of 0.85% saline solution was added to the resultant pellet. Conidial concentrations for each strain were determined using a hemocytometer and diluted accordingly to obtain a final concentration of 4.25 × 10^6^ conidia/mL that was used for subsequent assays.

The germination assay protocol was based on Clinical and Laboratory Standards Institute (CLSI) standards with modifications [42]. For each fungal strain, there were 3–4 controls and *A. striata* treatments. In each microcentrifuge tube, we placed 75 µL Roswell Park Memorial Institute medium (RPMI), 785 µL *A. striata* filtrate (ca. 90% total assay mixture), and 30 µL of fungal-conidial inoculum. Samples were incubated at 25 °C in the dark for 12–18 h depending on the strain [25,42]. Control assay tubes had the same contents, but ROH_2_O was substituted for *A. striata* filtrate. After incubation, assay tubes were centrifuged at 9300× *g* for 10 min, the assay media was then removed, and 0.5 mL of lactophenol-carbofuschin stain was added to tubes containing fungal pellets. The tube and its contents were mixed and centrifuged at 9300× *g* for 10 min. After centrifugation, the resultant supernatant was removed leaving a 0.05 mL solution containing the fungal pellet that was then mixed and mounted onto microscope slides, coverslips were then added and sealed with clear nail polish and left to dry before fungi were enumerated under the microscope. For quality control of contamination in the *A. striata* extract used in each experiment, 1 µL of the *A. striata* filtrate was plated onto PDA plates. These plates were incubated at both 36 °C and 25 °C. Germination assays were then repeated three times with each of the fungal strains demonstrating significant hyphal aberrations (Fo69, Fs02, and Pv19).

From the prepared germination assay slides, the number of germinated and aberrated hyphae was determined for the first 200 hyphae that were encountered on a given slide via raster pattern slide navigation. Frequency values were calculated as the quotient of the total number of germinated and aberrated hyphae divided by 200, multiplied by 100 to yield a percentage value. Hyphae were counted as having germinated if their germ tubes were equal to or greater than the diameter of swollen conidia from which they emerged. Hyphae were considered aberrated if there were any visible increases in their diameters at any given location on the hyphae that caused non-parallel cell walls. All quantification was performed via a light microscope (Zeiss, Thornwood, NY, USA) at 400× total magnification.

To quantitatively confirm increases in hyphal diameter, hyphal diameters at the sub-conidial and sub-apical regions of hyphae were measured via scanning electron microscopy (SEM). To understand whether increases in diameter are specific to hyphae or whether increases also occur in the parent cells (conidia), conidial diameters were also measured. Germination assays with Pv19, Pv06, Pv23, and *A. striata* were performed as described in the aforementioned germination assay protocol with the following modifications. After the first centrifugation post incubation, samples were sequentially washed with ROH_2_O and centrifuged each time to remove debris. Samples were then placed on 1% poly-L-lysine coated coverslips and vapor fixed with 1% OsO_4_ for 4 days and left to dry for another 4 days_._ After drying, the coverslips were mounted onto stubs and gold coated (90 nm of gold). Conidial diameters were measured at the widest area in the middle of each conidium. Due to field of view limitations at 5000× total magnification, we could not measure the midpoint region of hyphae. The measured sub-conidial region is the region extending less than ca. 1 µm from the conidium and corresponds to the nascent hypha. The measured sub-apical region corresponds to the hyphal location ~1 µm from the apical tip.

**Figure 1:**
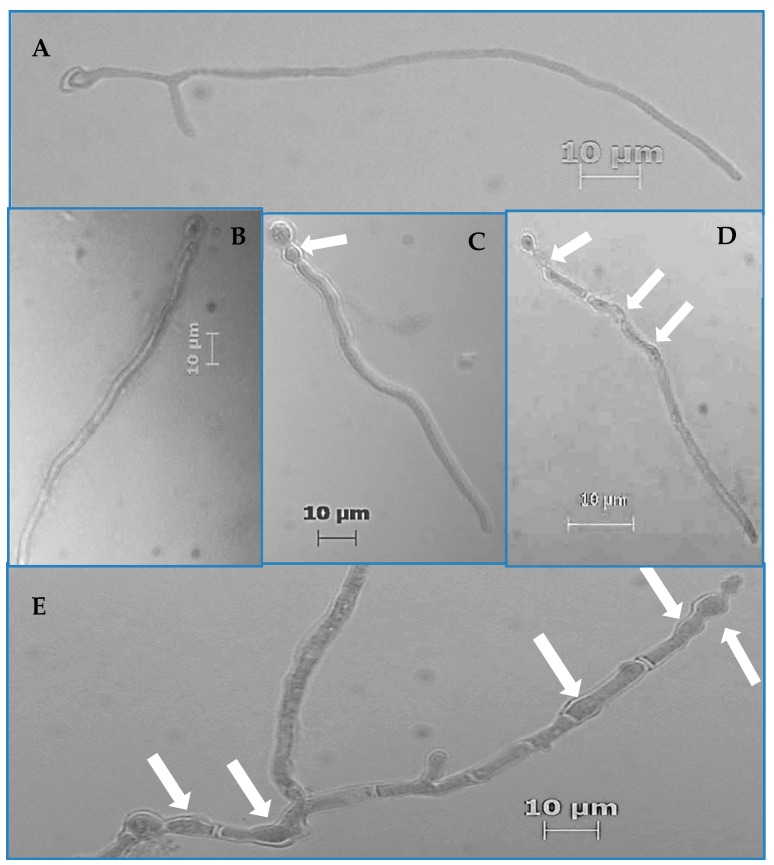
Examples of the various fungal morphologies encountered in germination and adhesion assays. (**A)** Reverse osmosis water (ROH_2_O) (control) treated fungi; (**B**–**E**) Different morphologies observed in *A. striata* treated Pv19; (**B**) swollen with parallel cell walls (SP), (**C**) swollen with non-parallel cell walls in the sub-conidial hyphal region (SNPSC), (**D**) swollen with non-parallel cell walls up to the midpoint hyphal region (SNPM), and (**E**) swollen with non-parallel cell walls throughout the hyphal length (SNPL). Arrows point to locations on hyphae where non-parallel cell walls are present.

For the adhesion assay, the aforementioned germination assay protocol was followed using three strains of *P. variotii* (Pv19, Pv06, and Pv23) and *A. striata*. Instead of microcentrifuge tubes, Lab-Tek two chamber slides (EW-01838-23, Cole-Parmer, Vernon Hills, IL, USA) were used. To aid fungal enumeration, 20 mm × 20 mm adhesive grids (GRID-1000, Diversified Biotech, Dedham, MA, USA), stickers with grids printed on them, were affixed to the bottom of each chamber slide. Only one chamber (the chamber furthest from the slide label area) was used. Once the fungal inoculum, RPMI, and *A. striata* or ROH_2_O (control) was added to the chamber slide, the mixture was gently swirled to mix the contents. Slides were incubated in the dark at 25 °C [25,42]. Replicates for each treatment were staggered by 30 min to allow time to count and wash each slide. Samples were removed from incubation after 18 h. The assay media including ROH_2_O or *A. striata* was carefully pipetted away and the incubation chamber was detached from the slide. The total number of fungi within each of the chosen 40 test quadrants on the grid was then counted. In addition to the total number of fungi present, we also recorded the total number of adhered fungi belonging to previously observed morphotype groupings (non-aberrated (NA), swollen with parallel cell walls (SP), swollen with non-parallel cell walls up to hyphal midpoint (SNPM), swollen with non-parallel cell walls throughout hyphal length (SNPL), and swollen with non-parallel cell walls in hyphal sub-conidial region (SNPSC) (Figure 1). We alternated between the 10× and 40× objectives to locate test quadrants and examine fungal morphology. After the pre-wash enumeration, slides were inserted into a modified slide holder (Lock Mailer^TM^ Microscope Slide Mailer and Staining Jar without Capinserts^TM^ #21096, Ted Pella, Waltham, MA, USA) and washed three times. To wash slides with uniform water flow, jars were modified by cutting 0.3 cm diameter holes in the center of the bottom of the jar and the lid [43,44]. Slide holder jars holding one slide were placed into a 4000 mL beaker that contained 3000 mL of ROH_2_O while a gloved finger was held over the hole on the jar lid. After the tube was submerged in water with 5 mm of space left between the jar and beaker bottom, the finger covering the hole on the jar lid was removed to allow water to fill the jar. Once the jar was filled with water, the finger was replaced back over the jar lid hole. With a finger still over the hole on the lid, the jar was moved over to a separate waste container, the finger was then removed from the lid hole to allow the water to drain from the slide holder and staining jar into a waste beaker. This washing step was repeated two more times for each slide. After the washing step, slides were patted dry on the bottom and the side of the slide with samples was stained with 10 µL lactophenol blue stain. A 22 mm × 22 mm coverslip was placed over the stained area and the same test quadrants that were counted in the pre-wash count were counted again, alternating between the 10× and 40× objectives. Cells on the left and bottom borders of a grid square were included in the total count for any given square while ones on the right and top borders of a square were excluded [43,44]. Results from treatments and controls (the number of conidia or hyphlets that adhered to slide 18 h post incubation) were compared to determine whether fungi with *A. striata* induced hyphal aberrations adhere to slides well. Results are reported as the percent adhesion, and for each test quadrant, the percent adhesion is calculated by dividing the post-wash count by the pre-wash counts times 100.

All statistical analyses were carried out in R [45]. Germination and aberration frequency data were analyzed using logistic regression. Germ tube length data were analyzed via ANOVA. Hyphal and conidial diameter data were analyzed using linear mixed-effects model via the lme4 package [46,47]. Adhesion assay results were analyzed using the Mann-Whitney Rank Sum test.

## 3. Results

**Figure 2:**
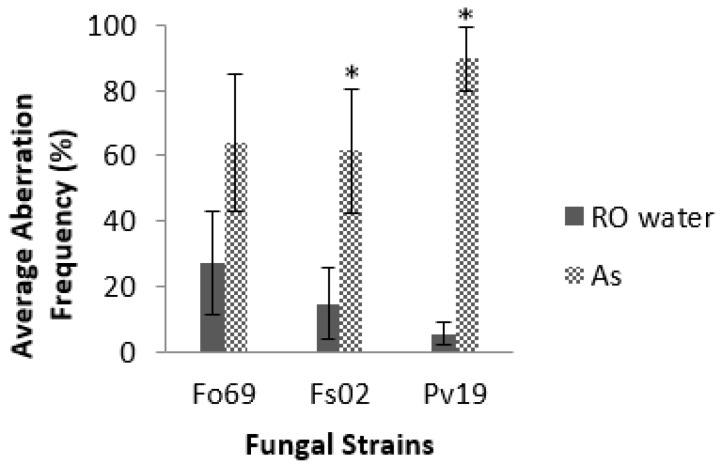
Effect of *A. striata* inner leaf gel on the aberration frequency of selected *F. oxysporum* (Fo69), *F. solani* (Fs02), and *P. variotii* (Pv19) strains in repeated germination assays. * Indicates statistical significance, error bars represent standard error of the mean, *n* = 200, *p*-value < 0.01.

Screening assays indicated that *A. striata* inner leaf gel filtrate decreased germination by 1.7% to 24.0% when compared to controls. These decreases were significant in one strain of *P. variotii* (Pv19), one strain of *F. solani* (Fs02), and two strains of *F. oxysporum* (Fo69 and Fo24) (*p* < 0.05, Table 2). Of the two *F. oxysporum* strains, Fo69 demonstrated the greatest decrease of 19.0%. *A. striata* also significantly increased germination frequency in the Fs53 strain of *F. solani* and two strains of *P. variotii* (Pv06 and Pv23) (*p* < 0.001, Table 2). When assays were repeated on Fo69 to verify results from initial screening experiments, Fo69 demonstrated a germination frequency of 92.0% ± 5.0% in controls and 83.0% ± 16.0% in treatments (*p* < 0.001). We also observed hyphal morphological aberrations characterized by increased hyphal diameters resulting in intervals of nonparallel regions of the hyphal cell wall as well as increased parent cell (conidium) diameter (Figure 1**C**–**E**). Of all the strains, Pv19, Fo69, and Fs02 exhibited the most significant average aberration frequencies of 96.0%, 37.0%, and 26.0%, respectively, when compared to controls (*p* < 0.05, Table 2). Repeated assays on Fo69, Fs02, and Pv19 had average aberration frequencies of 64.0% ± 21.0%, 62.0% ± 19.0%, and 90.0% ± 10.0%, respectively, in *A. striata* treated samples and 27.0% ± 16.0%, 15.0% ± 11.0%, and 5.0% ± 0.3%, respectively, in ROH_2_O treated samples (*p* < 0.001, Figure 2).

There was a significant increase in the parent cell diameter of *A. striata* treated Pv19 on all three days (*p* < 0.001, Table 3). Sub-conidial and sub-apical diameter measurements indicated an increase across all days (*p* < 0.001), except for the Day 1 sub-conidial measurements of Pv19 (*p* = 0.54, Table 3). Pv06 and Pv23 had a significant diameter increase in their sub-apical regions on all three days (*p* < 0.001, Table 3) and a significant increase in sub-conidial and conidial diameters (*p* < 0.001, Table 3) except for the Day 1 conidial diameter of Pv06 (*p* = 0.33, Table 3) and Day 1 sub-conidial diameters of Pv23 (*p* = 0.51, Table 3).

**Figure 3:**
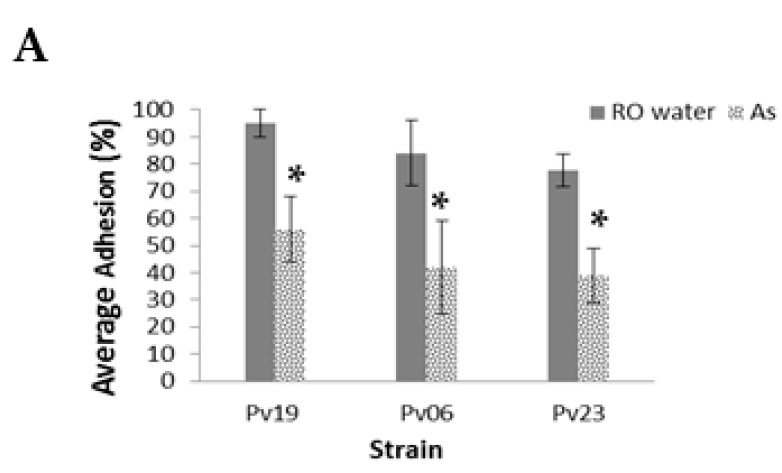

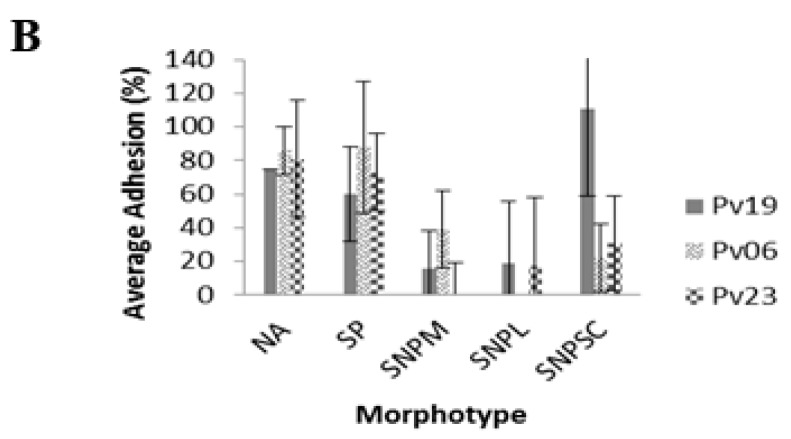
Average percent of adhered fungi in adhesion assays. (**A**) Average total percentage of adhered *P. variotii* strains (Pv19, Pv06, and Pv23) after treatment with *A. striata* (As) and reverse osmosis water (RO water) in adhesion assays that were repeated on three separate days with three replicates per day; (**B**) Average total percentage of the total adhered fungi per morphotypes (non-aberrated (NA), SP, SNPM, SNPL, and SNPSC) per *P. variotii* strain. * Indicates *p*-value < 0.001.

In *A. striata* treatments, the average total percent adhesion of Pv19, Pv06, and Pv09 was 5.0% ± 12%, 31.0% ± 13%, and 36.0% ± 24.0%, respectively, while controls had 95.0 ± 5.0%, 81.0% ± 15.0%, and 79.0% ± 10.0%, respectively, (*p* < 0.001, Figure 3A, see Table S1 for the number of fungi adhered to slides before the washing step). Of the total adhered fungi, we found that 75.0 ± 0.0%, 86.0% ± 13%, and 52.0% ± 35.0% of NA from treatment slides remained adhered to slides after the washing step for Pv19, Pv06, and Pv23, respectively. Pv06 controls had 84.0% ± 12.0% adhesion and *A. striata* treatments had 86.0% ± 13.0% adhesion among NA morphotypes. Fungi with SP morphology demonstrated 100.0% ± 0.0%, 84.0% ± 12.0%, and 73.0% ± 35.0% in controls while treatments demonstrated 60.0% ± 28.0%, 88.0% ± 33.0%, and 84.0% ± 23.0% for Pv19, Pv06, and Pv23, respectively (*p* < 0.001, Figure 3B, Table S2). The SNPM morphotype demonstrated 13.0% ± 23.0%, 0.0% ± 0.0%, and 0.0% ± 0.0% adhesion percentage in controls of Pv19, Pv06, and Pv23, respectively, while adhesion percentages in *A. striata* treatments were 16.0% ± 22.0%, 39.0 ± 31.0%, and 9.0% ± 19.0%, respectively. SNPL morphotypes demonstrated adhesion percentages of 0.0% ± 0.0% for both Pv19 and Pv06. There were no SNPL observed in the pre-wash count of Pv23. The SNPL adhesion percentages for *A. striata* treatments were 19.0% ± 37.0%, 0.0% ± 0.0%, and 17.0% ± 41.0% for Pv19, Pv06, and Pv23, respectively. In Pv06 and Pv23, the average number of adhered SNPSC in controls was 0.0% ± 0.0% and 1.0% ± 3.0% for Pv19. SNPSC demonstrated 111.0% ± 52.0%, 22.0% ± 20.0%, and 31.0% ± 28.0% for Pv19, Pv06, and Pv23, respectively (Figure 3B, Table S2).

## 4. Discussion

We initially identified strains of *F. solani* (Fs02, Fs20, Fs53), *F. oxysporum* (Fo69, Fo24, Fo57), and *P. variotii* (Pv19, Pv06, Pv23) via germination assays that exhibited sensitivity to treatment with *A. striata* in regards to germination. Repeated germination assays on Fo69 indicated a significant decrease in germination frequency. While *A. striata* inner leaf gel does cause differences in germination frequencies when compared to the controls of certain strains, the observed differences are marginal in respect to the prevention and treatment of opportunistic fungal infections. Due to the nature of mycelial formation, if at least one conidium can germinate, it can grow exponentially to form a mycelium and colonize hosts and/or surfaces. Although germination frequency decreases were marginal, we were able to confirm that *A. striata* caused a significant increase in the aberration frequency of Fs02 and Pv19 in repeated germination assays. While Fo69 demonstrated a significant increase in aberration frequency, the error bars on the graph did overlap for treatment and controls. This may be due to a possible day effect. Overall, Pv19 consistently demonstrated the highest increase in aberration frequency. The diameter increases associated with hyphal aberrations were confirmed via SEM measurements in three *P. variotii* strains. Significant increases in hyphal diameter were noted in the sub-apical regions on all three experiment days. Pv06 samples had a significant increase in the sub-conidial region on all three experiment days. However, Pv19 and Pv06 demonstrated sub-conidial hyphal increases on two of the experiment days. As discussed previously, it is difficult to get uniform conidial harvests, because, since fungal cultures are allowed to grow for three days before conidial harvest, the age of conidia present within any given culture may differ. Therefore, a day effect was observed when data was analyzed. In addition to the sub-apical and sub-conidial regions of hyphae, the conidia of *A. striata* treated Pv19 demonstrated a significant increase in diameter for all three experiment days. Pv06 and Pv23 demonstrated conidial increases on two experiment days. While conidia swell before germination, these conidial diameter increases were greater than those of the controls. Therefore, we can conclude that at least in Pv19, treatment with *A. striata* induces diffuse swelling. Similar diameter increases were observed in *Saprolegnia* treated with Congo red [48,49].

To understand whether hyphal aberrations cause a decrease in the ability of three *P. variotii* strains (Pv19, Pv06, Pv23) to adhere to substrate, we performed adhesion assays with Lab-Tek chamber slides. Adhesion assay results support our hypothesis that hyphal aberrations characterized by diameter increases leading to pronounced intervals of non-parallel cell walls along hyphae contribute to a decrease in the ability of fungi to adhere to substrate. The percent adhesion was higher in controls than in treatments for all three *P. variotii* strains with hyphal aberrations. Furthermore, when the total number of adhered fungi was separated into morphotypes, fungi with non-parallel cell walls, except for Pv19 SNPSC, consistently demonstrated lower adhesion percentages when compared to morphotypes with parallel cell walls, irrespective of whether morphotypes were on control or treatment slides. In Pv19 controls, SNPSC morphotypes had a percent adhesion of over 100%, because such results indicated that fungi with the SNPSC morphotype that were not present within test quadrants were displaced into the test quadrants after slides were washed three times. Since the SNPSC morphotype has non-parallel cell walls in the sub-conidial location, it has a reduced amount of surface area available to adhere to slides compared to morphotypes with parallel cell walls, but more surface area available for adhesion when compared to the SNPM and SNPL non-parallel cell wall morphotypes. SNPSC morphotypes could be displaced. However, because of the force from the washing step was not enough to displace them completely off the slide, they instead dislocated into neighboring quadrants, at least in the case of Pv19. Therefore, considering that SNPSC are able to be displaced, but due to the fact that aberrations are only in the sub-conidial area, the majority of the length of the hyphal cell wall is parallel and affords displaced hyphae more surface area for adhesion.

Non-parallel cell walls correlate with a decrease in hyphal surface area available for adhesion to substrates and morphotypes with the most regions of non-parallel cell walls having decreased adhesion frequencies. This phenomenon is evident when the interactions among fungi on the slides are considered. We noted instances where SNPSC, SNPM, and SNPL morphotypes appeared to be pinned onto slides by SP and NA morphotypes. Since SP morphotypes have the most readily available surface areas for adhesion, they have higher adhesion percentages. Pv19 treatment slides had at least four times more SP morphotypes then Pv06 and Pv23, leading to an increase in the likelihood of such interactions between individual Pv19 fungi. Moreover, comparison of the total number of adhered fungi in the pre-wash counts across the three strains indicates that Pv19 adheres better to surfaces than Pv06, which has relatively poor adhesion overall. The Pv19 morphotypes with parallel cell walls, NA and SP, showed slightly higher displacement when treated with *A. striata* than controls. However, when compared to morphotypes with non-parallel cell walls, the displacement of NA and SP is significantly lower. Furthermore, the SP morphotype, due to the fact that it is swollen with parallel cell walls, has more surface area readily available to adhere to surfaces than do all the observed morphotypes. Therefore, it is not surprising that SP morphotypes would demonstrate higher adhesion percentages then NA morphotypes.

While our results suggest that hyphal morphology plays a significant role in decreasing adhesion of three *P. variotii* strains, we must acknowledge that there are other factors that contribute to fungal adhesion. Mechanically, the non-parallel cell wall regions cause portions of hyphae to not be in contact with substrate. In order for hydrophobins to mediate adhesion, they have to be in contact with both fungal cell wall surfaces as well as substrate surfaces. Without contact to substrate, secreted hydrophobins are not able to mediate adhesion in those regions. Hyphal regions that are not in contact with substrate surfaces demonstrate similar adhesion capabilities as *B. bassiana* strains with deleted hydrophobin genes from Zhang et al. [50]. Zhang et al. linked a decrease in hydrophobins to a decrease in adhesion and a subsequent virulence [50]. Since hyphal aberrations cause a decrease in substrate hydrophobin interactions, our experiments have demonstrated a significant decrease in adhesion and we also anticipate a decrease in the virulence of fungi with *A. striata*-induced non-parallel cell walls. However, we do not expect the decrease to be of the same magnitude of that reported by Zhang et al. since fungi with hyphal aberrations also have hyphal regions that are able to maintain contact with the surface of substrate [50]. Furthermore, Kashina et al. note that although *F. velutipes* extract causes a decrease in fungal adhesion to epithelial cells, it failed to prevent fungi-induced damage to cells [36]. Therefore, further experiments need to be performed to determine whether *A. striata*-induced hyphal aberrations also correlate to a clinically significant decrease in *P. variotii*’s virulence.

Our findings connecting *A. striata*-induced hyphal aberrations to adhesion could potentially have many real-world applications. While glass slides do not mimic the irregular surfaces of cells, these experiments do serve as a foundation for future studies that would offer a clearer understanding of the possible real-world applications of *A. striata* gel. For example, if aberrated hyphae demonstrate a decrease in adhesion to corneal cells, *A. striata* inner leaf gel could be employed to help decrease the occurrence of opportunistic fungal infections such as keratitis. In this respect, the gel could be formulated into prophylactic eye drops so that any fungal conidia that has found its way into the ocular regions of immunocompromised individuals and managed to germinate would be prevented from adhering to ocular surfaces if their hyphae become aberrated. Several studies suggest that *A. barbadensis* and *A. arborescens* inner leaf gel extracts can be used in eye drop preparations to safely treat inflammatory disturbances and/or diseases of the cornea and other external ocular parts of the human eyes [51,52,53]. Furthermore, Wozniak and Padnuch found that *A. barbendensis* extract had no cytotoxic effects on human corneal cells [53]. Since *A. barbadensis* and *A. arborescens* inner gel were not toxic to human cells, it is likely that *A. striata* will also be safe when used on human cells. To confirm that *A. striata* is not cytotoxic, cytotoxicity tests should be performed against human corneal cells as well as epithelial cells. Furthermore, since *F. solani* and *F. oxysporum* are also emerging human pathogens and they exhibit increased hyphal aberration frequencies when treated with *A. striata* in germination assays, future studies should include germination assays on the *F. solani* and *F. oxysporum* strains to determine whether hyphal aberrations affect their ability to adhere to surfaces.

These findings may also prove useful in settings where fungal biofilm formation is difficult to combat. It can be difficult to completely remove fungal and/or bacterial biofilm from water systems as well as medical devices and/or instruments such as catheters because only the top layers of biofilm are washed off during the sanitation process, while the first colonizing microbes that form the bottom layer of biofilms are protected from being washed off [54,55]. To combat fungal biofilm formation on medical devices, fungi that are commonly encountered on such devices can be tested in germination assays to determine whether *A. striata* causes aberrations on their hyphae. If such fungi do demonstrate increased hyphal aberration frequencies, pilot studies can be performed to determine whether it would be feasible to coat medical devices colonized by tested fungi with *A. striata* inner leaf gel and possibly gel from other *Aloe* spp. to reduce the ability of the initial layer of colonizing fungi from being able to adhere to the devices permanently after sterilization and sanitation steps. Similarly, feasibility tests can also be performed on pipes within water systems to determine whether the routine flushing of pipes with *A. striata* inner leaf gel minimizes fungal biofilm formation. Minimizing fungal biofilm in the pipes of water systems is important for preventing pipe blockages [56]. Furthermore, if other filamentous fungi, particularly those molds found on shower tiles, also exhibit such aberrations after treatment with *A. striata*, *A. striata* may also be employed as a household cleaner or shower tile coating.

## 5. Conclusions

Although *A. striata* caused minimal significant decreases in germination frequencies, it did cause significantly large increases in the hyphal aberration frequencies of all screened strains. These aberrations are characterized by increased hyphal diameters that result in non-parallel cell walls at intervals along hyphae. In order to confirm and understand the extent of the increases in hyphal diameters as well as understand whether diameter increases also occur in conidia, randomly selected hyphae and associated conidia of three different *P. variotii* strains (Pv19, Pv23, and Pv06) were measured at three different locations via SEM. Conidial diameters were measured at the widest portion of conidia. Hyphal diameter measurements were acquired at two different locations: the sub-conidial and sub-apical hyphal regions. There was a significant increase in the parent cell diameter of *A. striata* treated Pv19 on all three days. Sub-conidial and sub-apical diameter measurements indicated an increase across all days, except for the Day 1 sub-conidial measurements of Pv19. Pv06 and Pv23 had a significant diameter increase in their sub-apical regions on all three days and a significant increase in sub-conidial and conidial diameters except for the Day 1 conidial diameter of Pv06 and Day 1 sub-conidial diameters of Pv23.

Adhesion assay results suggest that hyphal aberrations characterized by diameter increases resulting in intervals of non-parallel cell walls along hyphae significantly contribute to a decrease in the ability of fungi to adhere to substrate. The percent adhesion was higher in controls than in treatments for all three *P. variotii* strains. When the total number of adhered fungi was separated into morphotypes, fungi with non-parallel cell walls, except for Pv19 SNPSC, consistently demonstrated lower adhesion percentages when compared to morphotypes with parallel cell walls, irrespective of whether morphotypes were on control or treatment slides. These results indicate that *A. striata* inner leaf gel is a viable source for the discovery of new antifungal compounds that can target hyphal adhesion in fungi.

## Figures and Tables

**Table 1:** List of all the strains used in this research with their corresponding source.

Strain ID in This Paper	Organism	Source	Source Strain ID No
Fs02	*Fusarium solani*	ARS (NRRL)	NRRL 22402
Fs20	*Fusarium solani*	ARS (NRRL)	NRRL 22820
Fs53	*Fusarium solani*	ARS (NRRL)	NRRL 22153
Pv19	*Paecilomyces variotii*	ATCC	ATCC 22319
Pv06	*Paecilomyces variotii*	ATCC	ATCC 28806
Pv23	*Paecilomyces variotii*	ATCC	ATCC 16023
Fo69	*Fusarium oxysporu*	ARS (NRRL)	NRRL 25369
Fo57	*Fusarium oxysporu*	ARS (NRRL)	NRRL 25357
Fo24	*Fusarium oxysporu*	ARS (NRRL)	NRRL 26924

ATCC = American Type Culture Collection, ARS = United States Department of Agriculture Agricultural Research Service (formerly known as NRRL) Culture Collection.

**Table 2:** Summary of results from the initial screening of the effect of *A. striata* filtrate on the average germination and aberration frequencies of three strains each of *F. oxysporum* (Fo69, Fo57, and Fo24), *F. solani* (Fs20, Fs02, and Fs53), and *P. variotii* (Pv19, Pv06, and Pv23).

Strain	Germination Frequency (%)			Aberration Frequency (%)		
	Control	Treatment	∆	Control	Treatment	∆
Fo69	87.0 ± 12.0	67.0 ± 9.5 *	20.0	51.0 ± 10.0	89.0 ± 27 *	38.0
Fo57	50.0 ± 2.0	48.0 ± 10.0	2.0	84.0 ± 6.4	85.0 ± 20.8 *	1.0
Fo24	100.0 ± 2.0	93.0 ± 5.0 *	7.0	45.0 ± 37.0	65 ± 19 *	20.0
Fs20	53.0 ± 18.0	41.0 ± 36.0	12.0	22.0 ± 26.0	80.0 ± 29.0	58.0
Fs02	99.0 ± 3.0	69.0 ±	30.0	36.0 ± 22.0	61.0 ± 37.0 *	25.0
Fs53	54.0 ± 25.0	40.0 *	13.0	28.0 ± 26.0	60.0 ± 29.0 *	32.0
Pv19	100.0 ± 1.0	67.0 ± 24.0 *	−4.0	3.0 ± 2.0	95.0 ± 47.0 *	92.0
Pv06	71.0 ± 7.0	96.0 ± 2.0 *	25.0	2.0 ± 1.0	40.0 ± 51.0 *	38.0
Pv23	71.0 ± 15.0	81.0 ± 11.0 *	10.0	3.0 ± 1.0	90.0 ± 9.0 *	87.0

*N* = 200, ∆ denotes difference between control and treatments where positive change implies an increase and negative change value implies a decrease when compared to controls. Statistical significance is denoted where * *p* < 0.05.

**Table 3:** Average conidial, sub-conidial, and sub-apical diameter increases in *A. striata* treated Pv19, Pv06, and Pv23 when compared to controls. Experiments were performed with three replicates on three separate days.

Day	Observed Average Diameter Increase (µm)		
	Conidial	Sub-Conidial	Sub-Apical
Pv19			
1	0.65 ± 0.11 *	0.05 ± 0.09	0.82 ± 0.12 *
2	0.89 ± 0.15 *	0.49 ± 0.12 *	0.94 ± 0.12 *
3	0.62 ± 0.17 *	0.30 ± 0.10 *	0.43 ± 0.09 *
Pv06			
1	0.14 ± 0.14	0.37 ± 0.08 *	0.25 ± 0.08 *
2	1.12 ± 0.11 *	0.93 ± 0.14 *	1.24 ± 0.15 *
3	0.75 ± 0.14 *	0.86 ± 0.13 *	1.22 ± 0.13 *
Pv23			
1	0.00 ± 0.14	0.07 ± 0.11	0.29 ± 0.10 *
2	0.40 ± 0.15 *	0.54 ± 0.12 *	0.38 ± 0.08 *
3	0.83 ±0.19 *	0.49 ± 0.11 *	0.69 ± 0.09 *

* Denotes statistically significant increase, *p* < 0.001.

## Supplementary Materials

The following are available online at http://www.mdpi.com/2309-608X/4/1/23/s1, Figure S1: *A. striata* voucher (MU25872) deposited at the Willard Turrell Herbarium (Miami University, Oxford, OH), Table S1: Total number of *P. variotii* strains, Pv19, Pv06, and Pv23 adhered to control and treatment slides before slides were washed with RO water in adhesion assays where ∆ denotes difference between controls and treatments where positive change value implies an increase and negative change value implies a decrease, Table S2: Total number of adhered fungi for each morphotype after exposure to *A. striata* before slides were washed with RO water in adhesion assays for 3 strains of *P. variotii*, Pv19, Pv06, and Pv23. NA = non-aberrated, SP = swollen with parallel cell walls, SNPM = swollen with non-parallel cell walls up to the hyphal midpoint, SNPL = swollen with non-parallel cell walls throughout the hyphal length, SNPSC = swollen with non-parallel cell walls in the sub conidial hyphal region
