# Supplementary material for: Inner Leaf Gel of Aloe striata Induces Adhesion-Reducing Morphological Hyphal Aberrations
Source: J Fungi (Basel). 2018 Feb 8;4(1):23. doi: 10.3390/jof4010023 (PMC5872326; doi:10.3390/jof4010023)
Supplement: Supplementary file 1 [file jof-04-00023-s001.pdf]

Supplementary Materials

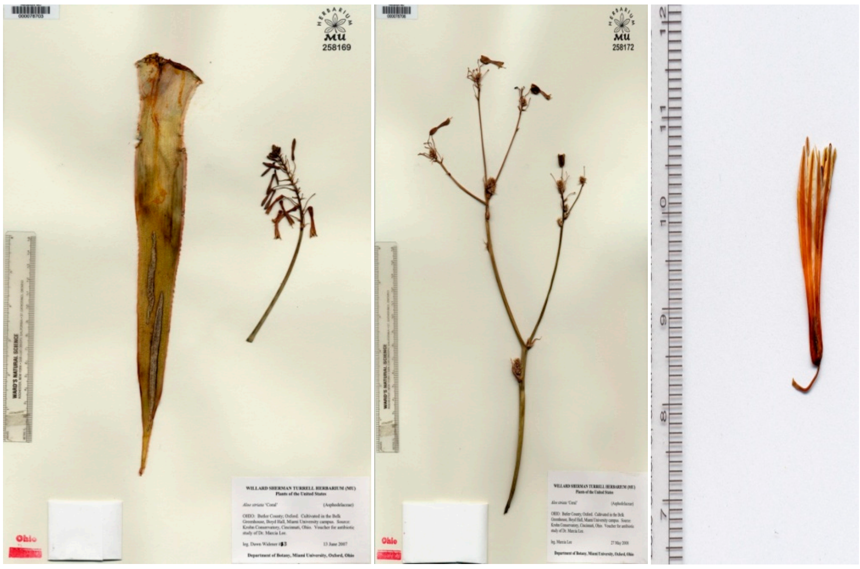

Figure S1. *A. striata* voucher (MU25872) deposited at the Willard Turrell Herbarium (Miami University, Oxford, OH).

Table S1. Total number of *P. variotii* strains, Pv19, Pv06, and Pv23 adhered to control and treatment slides before slides were washed with RO water in adhesion assays where Δ denotes difference between controls and treatments where positive change value implies an increase and negative change value implies a decrease.

Strain	Total fungi on slides before adhesion assay washing step		Δ
	Control	Treatment	
Pv19	5696.0	1348.0	−4348.0
Pv06	1742.0	422.0	−1320.0
Pv23	7236.0	1161.0	−6075.0

Table S2. Total number of adhered fungi for each morphotype after exposure to *A. striata* before slides were washed with RO water in adhesion assays for 3 strains of *P. variotii*, Pv19, Pv06, and Pv23. NA = non-aberrated, SP = swollen with parallel cell walls, SNPM = swollen with non-parallel cell walls up to the hyphal midpoint, SNPL = swollen with non-parallel cell walls throughout the hyphal length, SNPSC = swollen with non-parallel cell walls in the sub conidial hyphal region.

Strain	Pre-wash total adhered *A. strita* treated fungi of each morphotype (N)				
	NA	SP	SNPM	SNPL	SNPSC
Pv19	8.0	301.0	281.0	48.0	530.0
Pv06	6.0	7.0	15.0	3.0	272.0
Pv23	88.0	75.0	74.0	17.0	844.0
